# Structure of a truncated human GlcNAc-1-phosphotransferase variant reveals the basis for its hyperactivity

**DOI:** 10.1016/j.jbc.2024.107706

**Published:** 2024-08-22

**Authors:** Hua Li, Balraj Doray, Benjamin C. Jennings, Wang-Sik Lee, Lin Liu, Stuart Kornfeld, Huilin Li

**Affiliations:** 1 Department of Structural Biology, Van Andel Institute, Grand Rapids, Michigan, USA; 2 Department of Internal Medicine, Washington University School of Medicine, St. Louis, Missouri, USA

**Keywords:** mannose-6-phosphate targeting system, GlcNAc-1-phosphotransferase, human phosphotransferase, fly phosphotransferase, cryo-EM, structural biology, protein structure, enzyme catalysis, enzyme activity, *GNPTAB*, mucolipidosis II

## Abstract

Mutations that cause loss of function of GlcNAc-1-phosphotransferase (PTase) lead to the lysosomal storage disorder mucolipidosis II. PTase is the key enzyme of the mannose 6-phosphate (M6P) targeting system that is responsible for tagging lysosomal hydrolases with the M6P moiety for their delivery to the lysosome. We had previously generated a truncated hyperactive form of PTase termed S1S3 which was shown to notably increase the phosphorylation level of secreted lysosomal enzymes and enhance their uptake by cells. Here, we report the 3.4 Å cryo-EM structure of soluble S1S3 lacking both transmembrane domains and cytosolic tails. The structure reveals a high degree of conservation of the catalytic core to full-length PTase. In this dimeric structure, the EF-hand of one protomer is observed interacting with the conserved region four of the other. In addition, we present a high-quality EM 3D map of the UDP-GlcNAc bound form of the full-length soluble protein showing the key molecular interactions between the nucleotide sugar donor and side chain amino acids of the protein. Finally, although the domain organization of S1S3 is very similar to that of the *Drosophila melanogaster* (fruit fly) PTase homolog, we establish that the latter does not act on lysosomal hydrolases.

In vertebrates, lysosomal hydrolase targeting occurs mainly *via* the mannose 6-phosphate (M6P) pathway (1, 2). The hydrolases acquire the M6P tag during the process of maturation in the Golgi through two consecutive steps. First, GlcNAc-1-phosphotransferase (PTase) transfers GlcNAc-1-phosphate (GlcNAc-1-P) from UDP-GlcNAc to mannose residues on the high mannose glycans of the hydrolases (Fig. 1*A*), following which the GlcNAc moiety is removed by uncovering enzyme to generate the M6P monoester. Lysosomal hydrolases with the M6P tag bind with high affinity to M6P receptors (MPRs) and the receptor-enzyme complex is then packaged into clathrin-coated carriers for transport to the endo-lysosomal system (3, 4). Thus, PTase plays an essential role in the lysosomal biogenesis of vertebrates.

**Figure 1:**
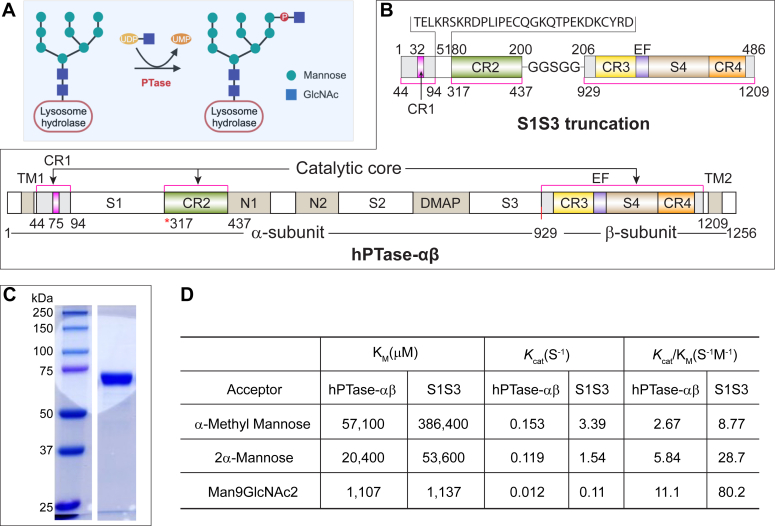
**Characterization of S1S3, a hyperactive truncation of hPTase-αβ.***A*, PTase catalyzes the transfer of GlcNAc-1-P to a mannose residue of a glycan chain in the lysosome targeting pathway. *B*, domain organization of human PTase-αβ (hPTase-αβ) and the truncated construct S1S3. The shared sequence between hPTase-αβ and S1S3 is shown in *light grey* and gradient color and marked by *pink lines*. *Numbers at the bottom* of S1S3 architecture correspond to amino acids in hPTase-αβ. The *red vertical line* at 929 marks the site-1 protease (S1P) cleavage site. *C*, coomassie Blue stained SDS-PAGE gel of the soluble S1S3 purified from the HPC4 affinity column. Twenty microliters of eluate was loaded on the gel. *D*, PTase activity of purified hPTase-αβ and S1S3 toward three *in vitro* acceptors. The data presented are the mean of two independent assays. CR1–CR4, Stealth conserved region 1 to 4; DMAP, DNA-methylation protein binding domain; EF, EF-hand domain; N1–2, Notch repeat domain; S1–4, Spacer domain 1 to 4; TM, transmembrane domain.

PTase is an α_2_β_2_γ_2_ heterohexamer encoded by two genes, *GNPTAB* and *GNPTG* (5, 6). The α and β subunits are transcribed and translated from the *GNPTAB* gene as a precursor molecule that is cleaved by site-1 protease (S1P) at Lys-928 to generate the individual subunits, a critical step for the activation of the molecule (7). These two subunits together comprise the catalytic core in addition to several other domains that mediate lysosomal hydrolase recognition and regulate enzymatic activity (Fig. 1*B*) (8, 9, 10, 11, 12). Three separate stretches in the primary sequence comprise the catalytic core; an N-terminal extension (NTE) to the first α-helix of the Spacer1 (S1) domain within which lies conserved region 1 (CR1). This is followed by conserved region 2 (CR2), and finally from the S1P cleavage site (Lys-928/Asp-929) to the C-terminal extension encoding conserved regions 3 and 4 (CR3 & CR4) (Fig. 1B) (12). Genetic mutations in human PTase (hPTase) cause the fatal lysosomal storage disorder (LSD) mucolipidosis II (MLII), or the less severe mucolipidosis III (ML III) depending on the nature of the mutation (13, 14).

LSDs include over 70 diseases arising from dysfunction of lysosomal hydrolases (15), such as Pompe and Fabry diseases caused by deficiency of lysosomal α-glucosidase and α-galactosidase (α-Gal), respectively (16, 17). While each LSD is rare, as a group they afflict many people around the world. Enzyme replacement therapy (ERT) is currently the main treatment option for LSDs (18). In our attempts to understand the role of the different domains of PTase for its function, and before its structure was elucidated, we had previously selectively deleted various regions of the protein resulting in the generation of a truncated hyperactive form of PTase termed S1S3 (11). In this minimal construct, the four conserved regions (CR1–CR4) common among Stealth domain proteins (19) were retained. The subsequently resolved structures of the human and zebrafish enzymes showed that these four regions formed the catalytic core (12, 20). Replacing S1 (233 aa) with a 29 aa linker sequence corresponding to the S1 region of the *Dictyostelium discoideum* PTase and deleting a large region from Notch 1 (N1) to the S1P cleavage site allowed for shortening of hPTase by 680 aa (Fig. S1). Compared with full-length hPTase, S1S3 was 20-fold more active *in vitro* and resulted in greater lysosomal hydrolase phosphorylation when transfected into cells (11). To gain a better understanding of the underlying mechanism for the increased phosphorylation, here we determined the cryo-EM structure of the purified soluble S1S3, which reveals complete conservation of the catalytic core similar to the full-length protein. We further determined the cryo-EM structure of the full-length PTase in complex with the nucleotide sugar donor UDP-GlcNAc to better understand substrate binding and catalytic mechanism. Interestingly, although the abbreviated domain architecture of the fruit fly PTase homolog is remarkably similar to S1S3, we demonstrate here that the fly enzyme does not act on lysosomal hydrolases.

## Results

### S1S3, a highly active truncation of hPTase

To investigate the molecular basis for the enhanced activity of S1S3 and to simplify structural determinations, a construct lacking both transmembrane domains was expressed as a soluble protein secreted from *Tricopulisia ni* (T. ni) cells and purified to homogeneity from the serum-free media (Fig. 1*C*). Lacking the cleavage site, the soluble S1S3 migrates as a single band on SDS-PAGE (Fig. 1*C*). Initial *in vitro* radiolabel assays showed soluble S1S3 retained PTase activity. Therefore, kinetic parameters were determined for both soluble S1S3 and soluble full-length hPTase (Fig. 1*D*). At saturating concentrations of [^3^H]UDP-GlcNAc, increasing amounts of three acceptor substrates were tested: α-methyl-mannoside (α-MM with a single mannose), the disaccharide 2-α-mannobiose (mannobiose), or Man9GlcNAc2 (Man-9) which most closely resembles the native glycan substrate. Similar to the relative activity seen with their membrane-bound counterparts in whole cell lysates (21-fold increase) (11), soluble S1S3 had a k_cat_ 22-fold faster than soluble hPTase for α-MM substrate (Fig. 1*D*). With mannobiose and Man-9 substrates, soluble S1S3 had k_cat_ values 13- and 9-fold greater than soluble hPTase, respectively (Fig. 1*D*).

### S1S3 is in the constitutively active configuration

**Figure 2:**
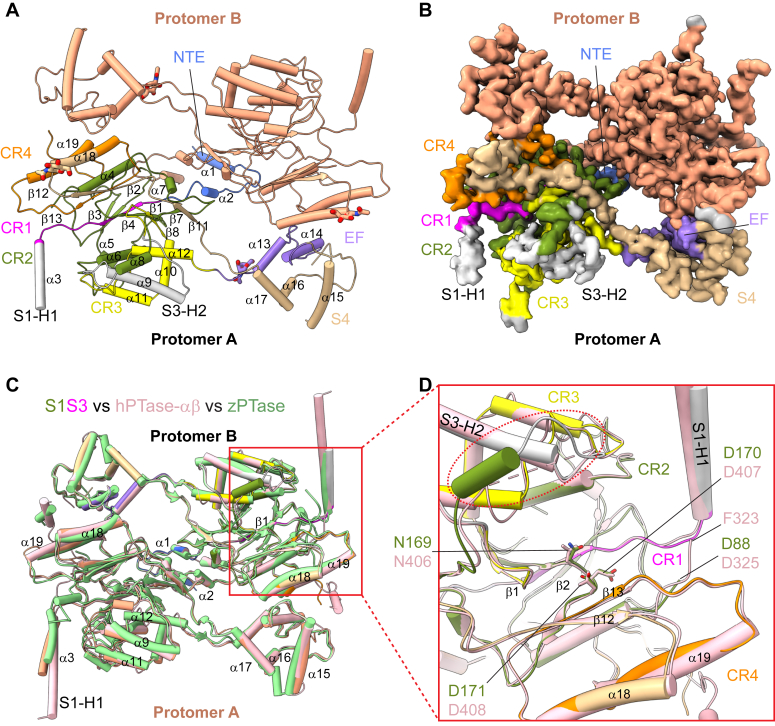
**Cryo-EM structure of the human S1S3 truncation.***A*, atomic model of S1S3 shown in the *cartoon*. Domains in Protomer A are colored as in Figure 1*B*. N-terminal extension (NTE) in shown in *blue*, and the two α-helices from S1 and S3 regions (S1-H1 and S3-H2) in *gray*. Protomer B is in *light coral*. The four resolved GlcNAc moieties at four glycosylation sites (two sites in each protomer) are shown in sticks. *B*, EM map of S1S3 in the same view as in *A*. *C*, superimposition of the structures of human PTase (hPTase)-αβ (*pink*, PDB ID 7S05), zebrafish PTase (zPTase, *green*, PDB ID 7SJ2) and S1S3 (color). S1S3 adopts a structure similar to hPTase-αβ and zPTase. *D*, close-up view of the catalytic pocket. The NDD motif is shown as *sticks*. The first resolved residue of CR2 is Phe-323 in hPTase-αβ and Asp-88 (corresponding to Asp-325 in hPTase-αβ) in S1S3, respectively. The *red dashed oval* highlights the GGSGG linker that replaces the N1-S3 sequence and directly connects CR2 to S3-H2 in S1S3. A *pink dashed line* represents the missing N1-S3 sequence in hPTase-αβ.

The structure of soluble S1S3 was determined by single-particle cryo-EM at an overall resolution of 3.4 Å (Fig. S2). Both 2D class averages and 3D maps show that S1S3 dimerizes similarly to PTase (Fig. 2, *A* and *B*) and has quite similar structural features as the catalytic core of PTase (12) (Fig. S2). The S1S3 structure comprises an NTE motif of two short α-helices, a large catalytic domain (the stealth domain) formed by the four conserved regions with a Rossmann-like GT-A fold, and a C-terminal small helix-bundle domain consisting of the EF hand and the three-helix S4 domain (Fig. 2, *A* and *B* and Table S1). While the four conserved regions (CR1–4) are distant from each other in primary sequence, they pack together. On the other hand, the EF hand and S4 are far away from CR3 and CR4, despite being connected by the two conserved regions in the primary sequence. Moreover, the EF hand interacts with CR4 of its counterpart subunit, thereby contributing to dimer formation (Fig. 2, *A* and *B*). This finding explains why mutations within the EF-hand cause disease (14) and inactivate the protein (21). S1S3 superimposes well on hPTase and zebrafish PTase (zPTase) with an RMSD of 0.63 and 0.75, respectively (Fig. 2*C*). The engineered linker replacing the S1 domain is still not observable (Figs. 1*B* and 2, *A* and *C*), while the short linker that connects CR2 and CR3 is evident (Fig. 1*B* and 2, *C* and *D*). S1S3 has essentially the same catalytic pocket with the catalytic NDD motif in the same orientation as other PTase structures (Fig. 2*D*). This structural merit indicates that S1S3 preserves all the catalytic features as seen in hPTase. However, while hPTase fluctuates between an active and inactive state due to an auto-inhibitory motif that moves in and out of the catalytic pocket corresponding with the closed and open nature of the S1 domain (12), S1S3 lacks both the S1 domain and the region from N1 to the S1P cleavage site that contains the auto-inhibitory motif (Figs. 1*B* and S1). Thus, S1S3 is likely locked in a constitutively active open state, consistent with its kinetic parameters (Fig. 1*D*).

### Donor substrate-bound form of hPTase

**Figure 3:**
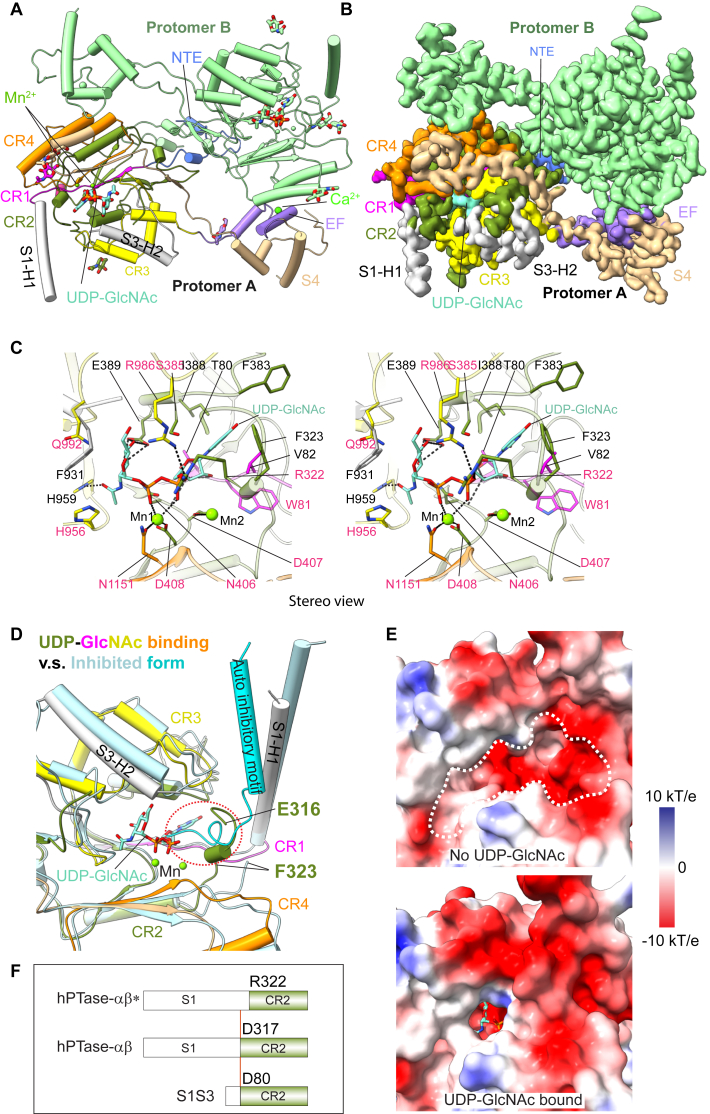
**Structure of hPTase-αβ bound to UDP-GlcNAc.***A*, *cartoon* view of the atomic model. The catalytic pocket of each protomer is occupied by one UDP-GlcNAc along with two Mn^2+^. Domains in protomer A are colored as in Figure 1*A*. The NTE is colored in *blue*, and two α-helices from the S1 and S3 regions (S1-H1 and S3-H2) are colored in *gray*. UDP-GlcNAc is shown in *sticks* and Mn^2+^ in *spheres*. Protomer B is shown in *pale green*. The eight resolved GlcNAc moieties at six glycosylation sites (three sites in each protomer) are also shown in *sticks*. *B*, EM map in the same view as in *A*. *C*, stereo view of the catalytic pocket formed by CR1-CR4. The donor substrate UDP-GlcNAc along with two Mn^2+^ (Mn1 and Mn2) bind to the pocket. UDP-GlcNAc and residues involved in the active pocket are shown in *sticks*, and Mn^2+^ in *spheres*. The enzyme main chain is in 90% transparent cartoon view. *D*, superimposition of the UDP-GlcNAc bound and the published auto-inhibited hPTase-αβ structures (PDB ID 7S06). The UDP-GlcNAc bound structure is colored as in (*A*). The auto-inhibited structure is in *pale cyan* except for the auto-inhibitory motif that is in *cyan*. The *dashed red oval* marks the C-terminal loop of the auto-inhibitory motif competing with UDP-GlcNAc and the upstream N-terminus of CR2 for the same space. The first resolved residue of CR2 with or without UDP-GlcNAc binding, namely Glu-316 and Phe-323, respectively, is highlighted. *E*, a close-up view of the electrostatic surface around the catalytic pocket of the active (*top*, PDB ID 7S05) and the UDP-GlcNAc-bound hPTase-αβ structure (*bottom*). The *dashed white shape* marks the unoccupied catalytic pocket in the active form. *F*, reassignment of the S1 and CR2 boundary. CR2 starts at Arg-322 in the previous assignment (hPTase-αβ∗). The CR2 N-terminal region starts at Asp-317 in the UDP-GlcNAc bound structure. Coincidentally, the S1S3 construct made (before the structures were available) started the CR2 at Asp-80, corresponding to Asp-317 in hPTase-αβ.

A short sequence (D317-R322) located immediately ahead of the conventional CR2 was not observed in the cryo-EM apo structures of either hPTase or the minimal zPTase that was modeled after S1S3 (Fig. 2*D*) (12, 20), despite the presence of the sequence in the zebrafish structure and in S1S3 (Fig. 1*B*). To determine if this region is necessary, we resolved the cryo-EM structure of hPTase in the presence of the nucleotide donor sugar UDP-GlcNAc at an average resolution of 2.9 Å (Fig. S3). The overall architecture shows no major change in the presence (Fig. 3, *A* and *B* and Table S1), or absence of UDP-GlcNAc (12), and resembles that of S1S3 (Fig. 2, *A* and *B*) and the engineered zPTase (20) (Fig. S4). This high-quality EM 3D map permits the entire UDP-GlcNAc molecule to be clearly observable (Fig. S5), and as expected, UDP-GlcNAc is in the conserved UDP-sugar binding pocket (Fig. 3, *A*–*C*) and binds in a manner highly similar to that in zPTase, with the exception that the two Mg^2+^ ions are replaced by two Mn^2+^ ions here (20) (Fig. S4).

The GlcNAc moiety H-bonds with Glu-389 *via* the neighboring O3 and O4 hydroxyls. In addition, it is in the proximity of Asn-406, Phe-931, His-956, His-959, and Arg-986 (Figs. 3*C* and S4). The α-phosphate of the UDP-GlcNAc forms a salt bridge with Arg-322 and Arg-986, and the α- and β-phosphates each contribute an oxygen atom to coordinate with Mn^2+^ (Mn1), as does Asp-408 (the last residue in the NDD motif) and Asn-1151 (Figs. 3*C* and S4). This feature is reminiscent of the GT-A fold seen in glycosyltransferases with a conserved DXD catalytic motif (22, 23). We further observe an additional Mn^2+^ (Mn2) without direct interactions with protein residues in the pocket (Fig. 3*C*). The closest residue to Mn2, Asp-407, is around 4 Å away, leading us to speculate this Mn^2+^ likely coordinates with water molecules. Indeed, in the X-ray crystal structure of the engineered zPTase with UDP-GlcNAc, this ion (Mg2) is fully coordinated by six water molecules forming an extensive hydrogen-bonding network with multiple side chains and main chain atoms (20). Also visible in our structure are the hydrogen bonds between the ribose O2′ and O3′ hydroxyl groups of the UDP-GlcNAc and Thr-80 and Asn-406, respectively. Moreover, Tyr-81, Val-82, Phe-323, Phe-383, Ser-385, and Ile-388 all lie within 3.5 Å of the uridine moiety (Fig. 3*C*). Consistent with this structure, the MLII-causing patient mutations, Tyr-81-Leu and Ser-385-Leu, result in loss of phosphotransferase activity (21, 24, 25, 26), while the missense mutations Asp-407-Ala, His-956-Arg, and His-956-Tyr are associated with MLIIIαβ (21, 27, 28, 29). In addition, we previously demonstrated that Asn-406-Ala and Asp-408-Ala abolished enzyme activity (12), while mutations of Arg-322-Gln, Glu-389-Leu, Arg-986-Lys, Gln-992-Leu, and Asn-1151-Ala were shown to notably diminish or abolish the transferase activity (20).

### S1S3, a minimal construct with GlcNAc-1-phosphotransferase activity

The extensive interactions described above produce a moderate conformational rearrangement around the active pocket and seem to indicate that UDP-GlcNAc pulls all these surrounding residues unto itself. Strikingly, the binding of UDP-GlcNAc extends the ordered structure of the N-termini of CR2 an additional seven residues, from Phe-323 to Glu-316 (Fig. 3*D*). This converts the deep and large pocket to a flat and shallow one (Fig. 3*E*). As a result, UDP-GlcNAc is largely enclosed in the active pocket, with only the β-phosphate accessible at the portal (Fig. 3*E*, lower panel). It is noteworthy that the well-ordered N-termini of CR2, along with the bound UDP-GlcNAc molecule, compete with the blade of the hockey stick-like inhibitory motif (Fig. 3*D*). Thus, the UDP-GlcNAc bound form of the enzyme locks hPTase in an active state, and the phosphoryl transfer can occur once an acceptor glycan chain is inserted into the pocket. Given the fact that the first resolved residue upstream of CR2 in the presence of UDP-GlcNAc is Asp-317 and this region (317-DISAS-321) is involved in the intact pocket formation (Fig. 3*D*), we propose that the N-termini of CR2 should be assigned to Asp-317 (Figs. 1*B* and 3*F*) and not Arg-322, which is the original assignment based on the sequence conservation of CR2 in bacteria (Fig. 3*F*, upper) (19). It is fortuitous that 317-DISAS-321 was retained in the highly active truncated variant S1S3 (Figs. 1*B* and 3*F*). Therefore, S1S3 may be the smallest truncation with full activity.

### Proposed catalytic mechanism

The UDP-GlcNAc binding pocket of PTase resembles a GT-A fold glycosyltransferase (12), such as EXTL2 which also utilizes UDP-GlcNAc as a donor substrate (30). However, PTase transfers GlcNAc-1-P to its acceptor substrate instead of just the sugar moiety, which is the case with the other glycosyltransferases. This likely entails a different catalytic mechanism. For our 3D EM mapping experiment, UDP-GlcNAc remained intact even after 6 h of incubation with hPTase and MnCl_2_. This indicates that UDP-GlcNAc hydrolysis is very slow in this system, unlike what happens in other glycosyltransferases (31, 32). Further, this suggests that PTase uses a one-step reaction mechanism without an enzyme-substrate intermediate.

**Figure 4:**
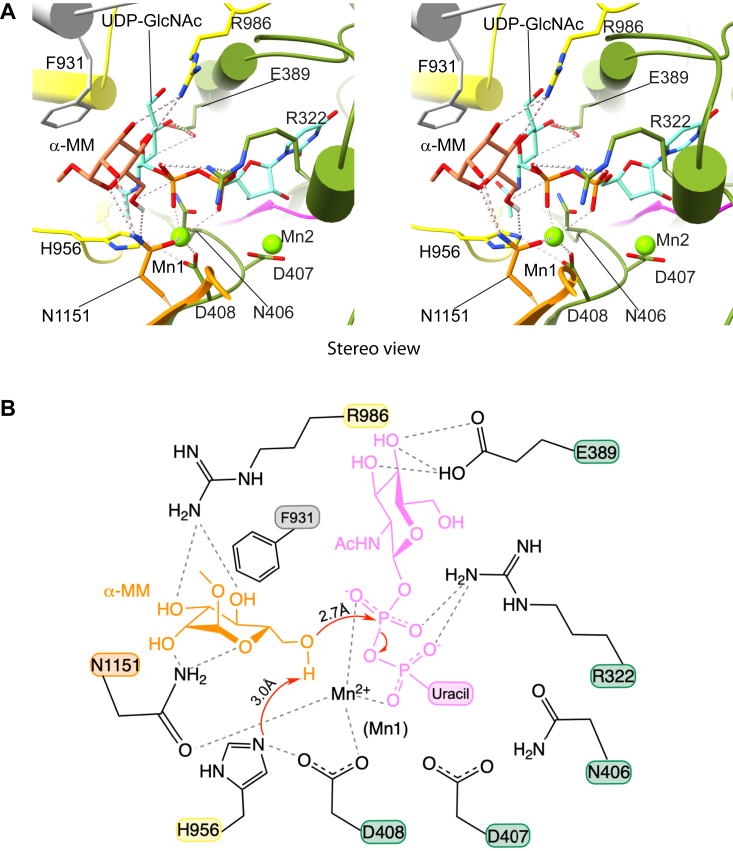
**Proposed catalytic model.***A*, stereo view of the modeled Michaelis complex of hPTase-αβ in the presence of donor UDP-GlcNAc and acceptor α-MM. The donor is from our experimental structure, but the binding mode of the acceptor is computed by Rosetta. The active site pocket is shown as a *cartoon*, with the substrates and key catalytic pocket residues shown in *sticks* and hydrogen bonds in *dashed gray lines*. The catalytic His-956 is reoriented by acceptor binding and by H-bonding with Asp-408. *B*, proposed catalytic mechanism. The reaction is initiated by His-956 deprotonating the O6 hydroxyl of α-MM. The O6 then launches a nucleophilic attack on the β-phosphate to form a new phosphoester bond, completing the transfer of GlcNAc-P to the mannose O6. The *red curved arrows* indicate either electron flow or cleavage of phosphoester bonds in UDP-GlcNAc. The residues are colored by their domain colors as in Figure 1*B*. See text for details.

The simple sugar acceptor substrate, methyl α-D-mannopyranoside (α-MM) was computationally docked into the pocket of the UDP-GlcNAc bound structure using Rosetta (33) (Fig. 4). Under this scenario, the UDP-GlcNAc binding pocket is largely unchanged except for four residues at the entrance of the pocket, namely Arg-322, Arg-986, Asn1151, and His-956, whose sidechains are reoriented (Figs. 3*C* and 4). As a result, Arg-322 forms a salt bridge with both α- and β-phosphate, and Arg-986 no longer H-bonds with GlcNAc but instead H-bonds with both the O3 hydroxyl and O4 hydroxyl (Figs. 3*C* and 4). Meanwhile, Asn-1151 H-bonds with O2 and the ring oxygen. Together, these four H-bonds tether α-MM to the catalytic pocket, and both the α- and β-phosphate are now stabilized by Arg-322 and Mn1 (Fig. 4). Importantly, the His-956 sidechain reorients and H-bonds with Asp-408 in the conserved ^406^NDD^408^ motif (12) and is positioned 3.0 Å away from the O6 hydroxyl (Fig. 4). Furthermore, these interactions position the α-MM O6 hydroxyl 2.7 Å away from the β-phosphate, well within the nucleophilic attack distance. Therefore, we propose that His-956 functions as the base to deprotonate the α-MM O6 hydroxyl. Upon deprotonation, the α-MM O6 launches a nucleophilic attack on the β-phosphate, leading to cleavage of the phosphoester bond between α- and β-phosphate and the formation of a new phosphoester bond between the α-MM O6 and the β-phosphate (Fig. 4*B*). This reaction mechanism is in agreement with the previous proposal based on the zPTase structure in which His-956 in human PTase was first identified to serve as the general base (20). This mechanism also explains why the highly conserved ^406^NDD^408^ motif is so important (12): in addition to coordinating UDP-GlcNAc, Asp-408 in this motif plays the critical role of orienting His-956 towards the acceptor O6 hydroxyl. Five key residues involved in the phosphotransfer reaction are highly conserved among vertebrates (20, 34), and mutations of these residues (Arg-322-Gln, Asp-408-Ala, His-956-Gln, Arg-986-Lys, and Asn-1151-Ala) were previously shown to either significantly diminish or abolish the phosphotransferase activity of the human enzyme (12, 20, 34).

### Functional analysis of the *Drosophila* homolog of PTase

**Figure 5:**
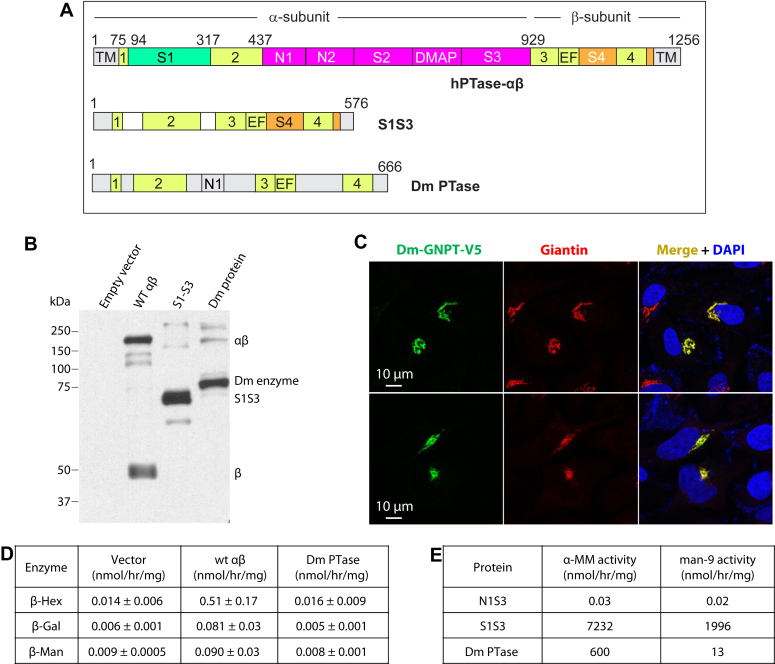
**Expression of *Drosophila melanogaster* PTase in HEK293T and *GNPTAB***^***−/−***^**HeLa cells.***A*, schematic of WT hPTase and the S1S3 variant in comparison with the putative Dm enzyme. *B*, immunoblot of mock (empty vector), WT PTase, the S1S3 variant, and the Dm enzyme expressed in *GNPTAB*^*−/−*^ HeLa cells; blot was probed with a monoclonal antibody against the V5 tag. Five micrograms of each cell lysate was loaded. The slower migrating minor bands (in the 150–250 kDa range) observed with S1S3 and the Dm enzyme likely represent a small amount of higher molecular weight aggregates since no signal was observed in the mock (empty vector) lane. *C*, immunofluorescence microscopy showing Golgi localization of the Dm enzyme in *GNPTAB*^*−/−*^ cells. *D*, β-Hex, β-Gal and β-Man activity following CI-MPR bead binding of extracts from *GNPTAB*^*−/−*^ HeLa cells expressing WT hPTase-αβ or the Dm PTase. The data are the mean ± SD of four independents. *E*, PTase activity of purified soluble S1S3 and Dm PTase using either α-MM (100 mM) or Man-9 (0.8 mM) as the acceptor. The purified soluble N1S3 completely lacks the catalytic domain and is used as a negative control. The data are the mean of two independent assays.

Although S1S3 is a synthetically engineered protein, the closest eukaryotic counterpart at the molecular level in nature is in fact a *Drosophila melanogaster* (fruit fly) protein that is the homolog of the mammalian PTase. Recently, the structure of this protein, henceforth referred to as Dm PTase, was solved by cryo-EM (35). Dm PTase resembles S1S3 in several respects, especially with regard to the organization of the conserved regions (Fig. 5*A*) and the absence of the S1P cleavage sequence. Thus, Dm PTase like S1S3 is a single polypeptide chain. At the amino-acid level, Dm PTase and S1S3 share 27% identity, with this value rising to 42% when similar residues are taken into consideration (Fig. S6). The fruit fly gene has one Notch domain, as opposed to two in full-length PTase, and lacks the DMAP domain. Neither the Notch nor the DMAP domains are present in S1S3. Moreover, the fruit fly as well as other insect species lack the gene for the γ subunit that mediates the recognition and binding of a subset of lysosomal hydrolases (35, 36). Further, the fruit fly homolog of the vertebrate MPR termed LERP lacks the key M6P recognition residues (37) and fails to bind phosphomannan (38). These findings raise doubts as to whether Dm PTase actually phosphorylates lysosomal hydrolases and if this organism even utilizes the M6P trafficking pathway. To date, there has only been one report of a *D. melanogaster* lysosomal enzyme that contains the M6P tag (39). However, microsomal membranes isolated from the Mediterranean fruit fly *Ceratitis capitata* do contain a protein that can use UDP-GlcNAc as the donor to transfer GlcNAc-1-P to α-MM, as well as to the glycan of the lysosomal purple acid phosphatase uteroferrin (40).

To further address this issue, we first asked if Dm PTase could rescue the phenotype of *GNPTAB*^*−/−*^ HeLa lacking endogenous PTase activity (9). As shown in Figure 5*B*, the level of expression of Dm PTase in these cells was comparable to wild-type (WT) PTase-αβ and S1S3 (Fig. 5*B*), and the Dm PTase is localized to the Golgi (Fig. 5*C*). However, Dm PTase, unlike WT PTase-αβ, could not restore M6P tagging of three lysosomal hydrolases that were tested, as determined by phosphorylated enzyme binding to cation independent-MPR (CI-MPR) affinity beads (Fig. 5*D*).

We next expressed and purified the secreted soluble forms S1S3 and Dm PTase from the media of T. ni insect cells and directly assessed their *in vitro* PTase activity using acceptors α-MM and purified Man-9. The purified N1-S3 domain of WT hPTase-αβ lacking any of the catalytic domains served as a background control. The purified Dm PTase in this case had 8% of the activity of S1S3 towards α-MM, but much lower activity was observed using the Man-9 glycan as the acceptor (Fig. 5*E*). These data demonstrate that the Dm PTase is capable of using UDP-GlcNAc as a nucleotide sugar donor and of transferring the GlcNAc-1-P moiety to α-MM in our *in vitro* assay. However, the efficiency of the transfer is poor relative to S1S3. His-956 is a critical residue within the UDP-GlcNAc binding pocket (20) and the equivalent histidine (His-375) is conserved in Dm PTase (Fig. S7, *A*–*D*). His-959, on the other hand, is mostly conserved in vertebrates but not in Dm PTase (Fig. S7, *A*–*D*) or other non-vertebrate species (19). We observed His-959 in hPTase to form a hydrogen bond with the GlcNAc moiety of UDP-GlcNAc. In contrast, the corresponding residue in Dm PTase, Phe-378 abolishes the H-bond and changes the electrostatic property of the donor UDP-GlcNAc binding pocket. To determine if His-959 is critical for activity, the equivalent residue in S1S3 (His-279) was mutated to phenylalanine (Fig. S7*D*). The His-279-Phe mutation caused a significant decrease in activity toward the three acceptors that were tested (51%, 56%, and 48% decreases, respectively, with α-MM (100 mM), mannobiose (10 mM), and Man-9 (0.34 mM)) (Fig. S7*E*). These results indicate that the H-bond between His-959 and the GlcNAc moiety is important and the loss of this interaction in the presence of phenylalanine at the equivalent position in Dm PTase contributes to the lower activity of the insect enzyme.

**Figure 6:**
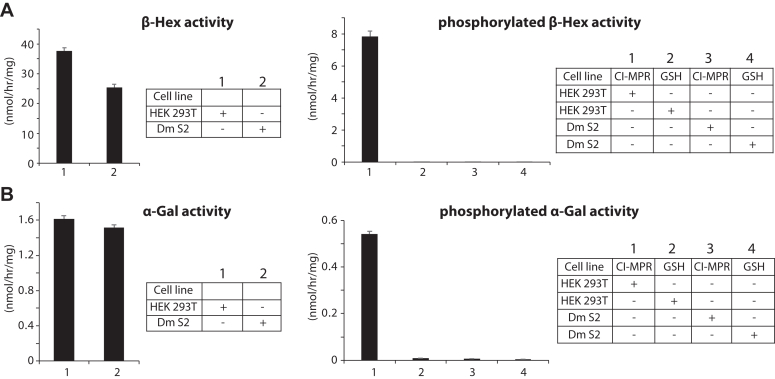
**Lysosomal enzyme activity in whole cell lysate *versus* CI-MPR bead pull-down of 293T and *D. melanogaster* S2 cells.***A*, β-Hex activity in HEK293T or S2 whole cell lysate (*left panel*) compared to the activity following CI-MPR bead binding (*right panel*). Activities are expressed as nmol of methylumbelliferone released per mg total protein per hour. *B*, α-Gal activity in HEK293T or S2 whole cell lysate (*left panel*) compared to the activity following CI-MPR receptor bead binding (*right panel*). The data shown are the mean ± SD for three independent assays.

We then asked if M6P-tagged lysosomal hydrolases were present in the fruit fly. We chose to look at two lysosomal hydrolases in fruit fly Schneider 2 (S2) cells, namely β-Hexosaminidase (β-Hex) and α-Galactosidase (α-Gal). The levels of endogenous β-Hex and α-Gal were first determined in whole cells extracts from HEK293T and S2 cells. Endogenous β-Hex levels were slightly lower in S2 cells compared to HEK293T cells while α-Gal levels were very similar (Fig. 6, *A* and *B*, left panels). However, unlike HEK293T cells, no M6P-tagged β-Hex or α-Gal was pulled down and detected with S2 cell lysates, as determined by CI-MPR bead binding (Fig. 6, *A* and *B*, right panels). Binding to glutathione (GHS)-agarose beads served as a negative control. These results demonstrate that β-Hex and α-Gal do not contain the M6P tag in fruit fly S2 cells.

**Figure 7:**
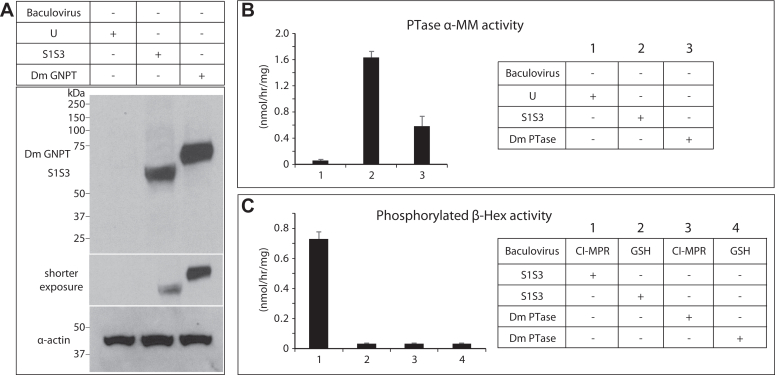
**Expression of human S1S3 and Dm PTase in S2 cells.***A*, immunoblot of uninfected (U) S2 cells, or S2 cells transduced with soluble S1S3 or soluble Dm PTase baculovirus. Fifty micrograms of cell lysate was loaded on the gel for detection of S1S3 and the Dm PTase using an anti-HA antibody. For detection of cytoplasmic actin, 10 μg of cell lysate was loaded. *B*, PTase activity using the artificial acceptor α-MM of uninfected S2 cell lysate (U), or S2 cell lysates expressing S1S3 or Dm PTase. *C*, β-Hex activity of uninfected S2 cell lysate (U), or S2 cell lysates expressing S1S3 or the Dm PTase following CI-MPR receptor bead binding. Activities are expressed as nmol of methylumbelliferone released per mg protein per hour. The data shown are the mean ± SD for three independent assays.

An examination of the β-Hex sequence from fruit fly revealed the presence of three N-linked glycosylation sites, compared to six in human β-Hex. We asked if these glycans on the fruit fly β-Hex could potentially be phosphorylated by overexpression of either S1S3 or Dm PTase. Using the already available baculoviruses for expressing soluble S1S3 and soluble Dm PTase in T. ni insect cells, we transduced the fruit fly S2 cells with the same baculoviruses to determine if we could achieve any protein expression under the control of the viral polyhedrin promoter. Although it has been reported that this promoter is only very weakly active or inactive in S2 cells (41, 42, 43), Western blot analysis revealed good expression of the Dm PTase under these conditions, while expression of S1S3 was much lower (Fig. 7*A*). Using equal amounts (50 μg) of whole cell extracts, PTase activity was measured using α-MM as the acceptor. The activity of S1S3 was approximately 3-fold higher than Dm PTase (1.64 ± 0.08 *versus* 0.59 ± 0.15) despite much greater expression of the latter (Fig. 7*B*). However, the activity detected with the Dm PTase was almost 10-fold higher than the background activity of uninfected S2 cells (0.06 ± 0.01). The CI-MPR bead pull-down assay was then used to assess if endogenous β-Hex in the S2 cells was phosphorylated by overexpression of the soluble S1S3 or the soluble Dm PTase. Indeed, S1S3 phosphorylated β-Hex in these cells, as shown by the activity recovered from receptor binding, but no activity above the background was observed with Dm PTase (Fig. 7*C*). Taken together, these results strongly indicated that while Dm PTase is likely a GlcNAc-1-P transferase, similar to bacterial stealth domain proteins (44), it is not involved in the M6P pathway.

## Discussion

In this study, the cryo-EM structure of the truncated hyperactive version of hPTase termed S1S3 was resolved at 3.4 Å. While attempts were made to image a complex of hPTase or S1S3 with Man_9_GlcNAc_2_, the native glycan presented by lysosomal hydrolases, no density of Man_9_GlcNAc_2_ was observed even when 50× Man_9_GlcNAc_2_ was added and incubated with protein sample overnight. One possible reason is that the affinity of Man_9_GlcNAc_2_ toward hPTase is still around 50-fold lower than most lysosomal hydrolases, ∼1 mM (Fig. 1*D*) *versus* ∼20 μM (45, 46). Moreover, as a result of the structural rearrangement that occurs in the presence of UDP-GlcNAc, the active pocket turns out to be shallow and flat. This implies that there is no specific binding pocket for the glycan chain. It is indeed documented that the Notch repeats, DMAP interaction domain, and the γ subunit of PTase play a key role in recognizing and binding lysosomal hydrolases (8, 9, 47). Therefore, protein-protein interaction, rather than protein-glycan interaction is more likely the recognition determinant. This is consistent with the fact that PTase modifies only lysosomal hydrolases over the hundreds of other glycoproteins that traverse the secretory pathway and present identical glycan chains, and also the finding that S1S3, lacking the recognition domains, loses some specificity and modifies non-lysosomal proteins with N-glycans (11). In the process of phosphoryl transfer, lysosomal hydrolases are recognized and bind to PTase *via* the Notch repeats and/or DMAP interaction domain, following which the glycan is orientated to the UDP-GlcNAc binding pocket to accomplish the reaction. Analysis of the structure of PTase in complex with a lysosomal hydrolase may facilitate the understanding of this process.

S1S3 is an engineered enzyme with only the essential catalytic core needed to produce lysosomal hydrolases with high levels of the M6P marker to enhance their uptake in a clinical setting (11). Lacking the regulatory and recognition domains permits the UDP-GlcNAc binding pocket of S1S3 to have constant access to the glycan chain of acceptor proteins, thereby enhancing its phosphoryl transfer efficiency, although some non-lysosomal proteins are also modified with the M6P tag in the process (11). Although this may not be desirable, the ability of S1S3 to highly phosphorylate a secreted lysosomal hydrolase to allow for more efficient cross-correction could potentially outweigh the disadvantage. Another advantage of S1S3 is its reduced size, being less than half the size of full-length hPTase-αβ. This makes S1S3 more amenable to adeno-associated virus (AAV)-mediated gene therapy for treatment of patients with MLII/III, since it is increasingly difficult to package larger length cDNA into the AAV capsid. In fact, the efficiency of expression of reporter vectors in excess of 5 kb is substantially reduced relative to reporter vectors with genomes <5 kb in length (48).

Therapy development can certainly benefit from a reliable disease model. Since *D. melanogaster* is a highly tractable genetic system, several LSD models have been developed in this model organism (49, 50, 51). Dm PTase shares a conserved fold with the catalytic core of hPTase (35). In terms of domain organization, Dm PTase is more similar to S1S3 and remarkably smaller than hPTase. Moreover, like S1S3, it does not require maturation by S1P cleavage, and with a shorter S1 domain, a single Notch repeat, and an EF-hand, it is not unreasonable to assume that it performs the same function as S1S3 (35). However, our results show that while Dm PTase can transfer GlcNAc-1-P to α-MM *in vitro*, it lacks GlcNAc-1-phosphotransferase activity *in vivo* towards the two lysosomal hydrolases that were tested in this study. Moreover, β-Hex in fruit fly S2 cells acquires the M6P tag when S1S3 is overexpressed in these cells but not Dm PTase. While a few lysosomal enzymes in fruit fly may contain phospho-residues on their high mannose oligosaccharides (39, 40), the lack of a receptor that would bind to M6P-tagged enzymes supports our hypothesis that fruit fly does not utilize the M6P targeting system for transport of hydrolases to the lysosome. It has been reported that the secreted glycoproteins vitellin and vitellogenin from other insect species have phosphosugar residues on their glycan chain (52, 53). Hence, it is tempting to speculate that these secreted glycoproteins and not lysosomal hydrolases are the true substrates of the Dm PTase. In this regard, precaution should be taken with the *Drosophila* models, especially for those LSDs caused by deficiency of PTase and lysosomal hydrolases.

## Experimental procedures

### Reagents and antibodies

UDP-[^3^H]GlcNAc (20–45 Ci/mmol) was purchased from PerkinElmer. Mouse anti-V5 monoclonal antibody (Cat #46-0705) was obtained from Thermo Fisher while anti-giantin rabbit polyclonal antibody (Cat # 924302) was from BioLegend. Anti-HA rabbit monoclonal antibody (Cat #C29F4) was from Cell Signaling and anti-α-actin rabbit polyclonal antibody (Cat #ABT1487) was from Millipore-Sigma. Methyl α-D-mannopyranoside (α-MM) and 2α-mannobiose (mannobiose) was from Millipore-Sigma. Man9GlcNAc2 (Man-9) was isolated from soybean agglutinin prepared by affinity chromatography as previously described (54).

### DNA constructs

The full-length human GlcNAc-1-phosphotransferase-V5/His construct in pcDNA6 as well as S1S3-V5/HispcDNA6 have been described (11, 21). The construct with the mutation in the UDP-GlcNAc binding pocket (His-959-Phe) was generated by Quick-Change mutagenesis, and DNA sequencing was performed to ensure that only the desired site was mutated. The *D. melanogaster* cDNA was made as a synthetic construct with nucleotides encoding the V5 epitope at the 3′ end in the vector pcDNA3 (GenScript).

For expression in insect cells, the cDNA of the human S1S3 and the Dm PTase without the N- or C-terminal transmembrane domains and cytosolic tails were cloned into the vector pFastBac1 (Invitrogen). To ensure the secretion of these proteins into the media, a DNA fragment encoding the signal sequence (SS) of honeybee melittin was cloned upstream and in frame with the cDNA sequences. Nucleotides encoding the hemagglutinin epitope (HA) and anti-Protein C epitope (HPC4) were appended to the 5′ end (downstream of the SS cleavage site) and 3′ end of the cDNAs, respectively.

### Cell lines

HEK293T and parental HeLa (ATCC), and *GNPTAB*^*−/−*^ HeLa cells (9) were maintained in DMEM containing 0.11 g/l sodium pyruvate and 4.5 g/l glucose, supplemented with 10% (vol/vol) FBS, 100,000 U/l penicillin, 100 mg/l streptomycin and 2 mM L-glutamine. Sf9 and T. ni cells were grown in serum-free conditions with SF900 II SFM and Express Five SFM, respectively. Both serum-free insect cell media were purchased from Invitrogen. *D. melanogaster* S2 cells were maintained in SF900 II SFM.

### Expression and purification of soluble S1S3 and Dm PTase

For expression in insect cells, S1S3pFB1 and DmPTasepFB1 were transformed into *E. coli* DH10Bac-competent cells to generate recombinant bacmids per manufacturer’s protocol (Invitrogen). Bacmid DNAs were prepared and transfected using Cellfectin II (Invitrogen) into Sf9 cells to produce recombinant baculoviruses that were amplified for two rounds. For protein expression, baculovirus was added to T. ni cells and 3 days post-infection, the soluble S1S3 and Dm PTase secreted into the serum-free media were collected and purified on an HPC4-agarose matrix (Sigma) according to the manufacturer’s protocol. Briefly, the equilibration and binding steps were performed using a buffer containing 20 mM Tris-HCl pH 7.5, 100 mM NaCl, and 1 mM CaCl_2_. The same buffer was used for the wash step except that the NaCl concentration was increased to 500 mM. The proteins were eluted in 1 ml fractions in a buffer containing 20 mM Tris-HCl pH 7.5, 100 mM NaCl, and 5 mM EDTA.

### CryoEM grid preparation and data collection

Quantifoil R2/1 300 mesh copper grids were freshly glow-discharged. Cryo-EM grids were prepared in an FEI Vitrobot Mark IV, with chamber humidity set to 95% and temperature set to 6 °C. Three microliters of the purified S1S3 at 0.8 mg/ml were applied on freshly glow-discharged Quantifoil grids, then the EM grids were blotted for 3 s by a piece of Whatman 595 filter paper with blotting force set to 2. The EM grids were then plunged into liquid ethane cooled by liquid nitrogen and stored in a liquid nitrogen dewar. To study the UDP-GlcNAc bound hPTase structure, 10 mM UDP-GlcNAc and 10 mM MnCl_2_ were added into 0.1 mg/ml hPTase and incubated for 6 h at 4 °C before cryo-EM grid preparation.

We used the same strategy to collect cryo-EM data for both S1S3 and UDP-GlcNAc bound hPTase. Movie micrographs were automatically collected on a Gatan K3 camera with SerialEM on an FEI Titan Krios microscope operated at 300 kV. The magnification was set to 105k×, corresponding to the pixel size of 0.828 Å at the specimen level. The objective lens defocus values varied from −1.0 to −1.6 μm. Each micrograph was recorded in a 60-frame movie with a total dose of 60 electrons per Å^2^ and a total exposure time of 1 s.

### Image processing

The S1S3 dataset was processed using CryoSPARC-v4 (55). The raw movies were motion corrected with Patch Motion Correction followed by contrast transfer function estimation and correction in Patch CTF. Based on the CTF quality, 13,219 micrographs were selected for further processing after Manually Curate Exposures. Template picking followed by 2D classification was carried out to produce initial particles for TOPAZ training and subsequent TOPAZ particle picking (56). Particles were extracted using a box size of 300 pixels. Particles belonging to 2D classes that lacked fine structural details were removed. Duplicated particles from different particle-picking methods were also removed. A total of 956,503 particles were retained and subjected to 3D classification. Particles in the dominant and best-quality 3D classes were selected for one more round of 3D classification. Finally, 275,102 particles were kept and used for 3D reconstruction and 3D refinement, leading to a 3D map at 3.4 Å resolution based on the cutoff value of 0.143 in the gold standard Fourier shell correlation curve.

A total of 24,130 movie micrographs were acquired for the UDP-GlcNAc bound hPTase complex. Sample drift in each movie micrograph was estimated and corrected by MotionCorr2 (v2.15)(57), and the contrast transfer function (CTF) was estimated and the effect was corrected by CtfFind-4.1.10 (58). Twenty two thousand five hundred nine micrographs with good particle contrast and CTF quality were imported into CryoSPARC-v4 (55) for further processing. Template picking followed by 2D classification was performed to generate particles for TOPAZ training and TOPAZ picking (56). The particles were extracted using a box-size of 320 pixels. After three rounds of 2D classification and duplicate removement, 1,965,813 particles belonging to 2D classes with fine structural features were imported to Relion-4.1 (59). Two rounds of 3D classification were carried out on this dataset, and 876,204 particles in the best 3D class were used for 3D reconstruction and refinement resulting in a 3D map at 2.9 Å resolution.

### Model building, refinement, and validation

For modeling the S1S3 EM map, the structure of hPTase in active form (PDB ID 7S05 (31)) was first rigid-body docked into the 3D map using ChimeraX-1.6.1, and the residues were renumbered according to the S1S3 sequence. Missing residues were manually added, and all residues were individually inspected and adjusted in COOT (60). Then, the derived model was refined by real-space refinement using Phenix (61).

For modeling the EM map of the UDP-GlcNAc bound hPTase, the active form of hPTase core (PDB ID 7S05) was used as an initial model and rigid-body docked into the 3D map by ChimeraX-1.6.1 (62). UDP-GlcNAc and Mn^2+^ were manually built into the EM densities in COOT (60). The derived model was refined using a real-space refinement module by Phenix (61) and manually adjusted in COOT (60).

Both S1S3 and the UDP-GlcNAc bound models were validated using MolProbity (63). To avoid overfitting, the Fourier shell correlations of the final model against the EM map and the two halfmaps (halfmap1 and halfmap2) were calculated. The structural figures were prepared using ChimeraX-1.6.1 (62).

### Molecular docking

The simple sugar α-MM, an acceptor analog, was docked into the catalytic pocket of the structure of the UDP-GlcNAc bound hPTase using RosettaLigand (33). In pre-docking preparation, UDP-GlcNAc was deleted from the pocket followed by hydrogen addition. Six conformers of α-MM were generated using Frog2 online server (64). Then the ligand parameter file was prepared using Rosetta script “molfile_to_params.py”. Finally, one conformer of α-MM was manually docked into its binding pocket in ChimeraX-1.6.1, to generate a starting model. One hundred docked poses were generated by RosettaLigand docking and manually inspected in ChimeraX-1.6.1 (62). The sketch of the catalytic mechanism was illustrated using ChemDraw 20.1.

### Transduction of *D. melanogaster* S2 cells with S1S3 or Dm PTase baculovirus

S2 cells growing in SF900 II serum-free, protein-free medium were transduced with the same baculoviruses used for expressing soluble S1S3 and soluble Dm PTase in T. ni cells. Cells were collected 72 h post-infection and processed for Western blotting and PTase α-MM activity or β-Hex activity.

### PTase activity assay

The protocol for measuring PTase activity was adapted from Lee *et al.* (65) with some modifications. Essentially, HEK293T cells were transfected at 50 to 60% confluency in six-well plates with a total of 2 μg of the indicated plasmid DNA and 3 μl jetOPTIMUS transfection reagent according to the manufacturer's protocol. Two days post-transfection, the cells were harvested and lysed in 150 μl of TBST (25 mM Tris-Cl, pH 7.4, 150 mM NaCl, 1% Triton-X 100, and protease inhibitor cocktail). Whole cell lysate (50 μg) was assayed in a final volume of 50 μl in buffer containing 50 mM Tris (pH 7.4), 10 mM MgCl_2_, 10 mM MnCl_2_, 2 mg/ml BSA, 2 mM ATP, 75 μM UDP-GlcNAc, and 1 μCi UDP-[^3^H] GlcNAc, with 100 mM α-MM as acceptor. The reactions were performed at 37 °C for 1 h, quenched with 950 μl of 5 mM EDTA (pH 8.0), and the samples applied to a QAE-Sephadex column (1 ml of packed beads equilibrated with 2 mM Tris, pH 8.0). The columns were washed twice, first with 3 ml of 2 mM Tris (pH 8.0), and then with 2 ml of the same buffer. Elution was performed twice, first with 3 ml of 2 mM Tris (pH 8.0) containing 30 mM NaCl, then once again with 2 ml of the same buffer. Eight milliliters of EcoLite scintillation fluid (MP Biomedicals) was then added to the vials, and the radioactivity in the collected fractions was measured using a Beckman LS6500 scintillation counter. All CPM values that were obtained after subtracting buffer-only background were converted to pmol of GlcNAc-phosphate transferred to α-MM per hour per mg total protein lysate (pmol/h/mg).

### CI-MPR bead pull down and lysosomal enzyme assay

Soluble bovine cation-independent mannose 6-phosphate (CI-MPR) was purified from fetal bovine serum as previously described (66). The purified CI-MPR was conjugated to CNBr-activated Sepharose 4B (Millipore-Sigma) as per the manufacturer's instructions. For lysosomal enzyme assays, 600 μg of whole cell lysates (prepared from transfected or untransfected HEK293T cells, or from uninfected or transduced *D. melanogaster* S2 cells) were incubated with equivalent amounts of the CI-MPR affinity beads for 2 h at 4 °C, washed twice with cold TBS, then assayed for the bound lysosomal enzymes. Briefly, β-hexosaminidase (β-Hex), and α-galactosidase (α-Gal) were assayed in a final volume of 50 μl with 5 mM 4-methylumbelliferyl(MU)-N-acetyl-β-d-glucosaminide (Sigma-Aldrich), and 5 mM 4-MU-α-d-galactopyranoside (Calbiochem), respectively, in 50 mM citrate buffer containing 0.5% Triton X-100 (pH 4.5) at 37 °C. Reactions were stopped after 1 h by addition of 950 μl of 0.4 M glycine-NaOH (pH 8.0). Samples were vortexed and spun down briefly, and the supernatant transferred to a round-bottomed glass tube. The fluorescence was then measured using a TURNER Model 450 Fluorometer (Barnstead Thermolyne Corporation), with excitation and emission wave lengths of 360 and 450 nm, respectively.

### Immunofluorescence microscopy

To visualize the subcellular localization of the Dm PTase, HeLa cells were transfected with the cDNA using the jetOPTIMUS transfection reagent. The cells were fixed 24 to 36 h post-transfection with 4% formaldehyde (Sigma-Aldrich) for 10 min, permeabilized and blocked with PBS containing 0.4% (v/v) Triton X-100 and 2% IgG free BSA (Jackson ImmunoResearch) for 1 h, and then probed with the indicated combinations of antibodies in PBS containing 0.1% Triton X-100 and 0.5% BSA. The processed cells were mounted in a ProLong Gold antifade mounting medium (Life Technologies), and the images were acquired with an LSM880 confocal microscope (Carl Zeiss Inc). Images were analyzed by Image J software (Fiji).

### Immunoblotting

Proteins resolved by SDS-PAGE under reducing conditions were transferred to nitrocellulose membrane and detected with antibodies as described in the figure legends. The indicated amounts of whole cell extract were loaded on the gels.

## Data availability

The 3D cryo-EM maps of S1S3 truncation at 3.4 Å average resolution and hPTase complexed with UDP-GlcNAc at 2.9 Å average resolution have been deposited in the Electron Microscopy Data Bank under accession codes EMD-44512 and EMD-44511, respectively, and their associated atomic models have been deposited in the Protein Data Bank under accession codes 9BGG and 9BGF, respectively. All data are available from the authors upon reasonable request.

## Supporting information

This article contains supporting information.

## Conflict of interest

The authors declare that they have no conflicts of interest with the contents of this article.
