# Supplementary material for: Structure of a truncated human GlcNAc-1-phosphotransferase variant reveals the basis for its hyperactivity
Source: J Biol Chem. 2024 Aug 22;300(9):107706. doi: 10.1016/j.jbc.2024.107706 (PMC11418123; doi:10.1016/j.jbc.2024.107706)
Supplement: Supplemental Tables and Figures [file mmc1.docx]

**Table S1. Data collection, processing, model refinement and validation statistics**

|  | **hPTase with UDP-GlcNAc**  (PDB ID 9BGF)  (EMD-44511) | | **S1S3 truncation**  (PDB 9BGG)  (EMD-44512) |  |
| --- | --- | --- | --- | --- |
| **Data collection and processing** |  |  |  |  |
| Microscope | Titan Krios (FEI) | | Titan Krios (FEI) | |
| Magnification | 105,000 | | 105,000 | |
| Voltage (kV) | 300 | | 300 | |
| Electron exposure (e^-^/Å^2^) | 60 | | 60 | |
| Defocus rang (μm) | 1.0-1.6 | | 1.0-1.6 | |
| Pixel size (Å) | 0.828 | | 0.828 | |
| Symmetry imposed | C1 | | C1 | |
| Initial particle images (No.) | 2,859,876 | | 2,249,987 | |
| Final particle images (No.) | 876,204 | | 275,102 | |
| Map resolution (Å) | 2.9 | | 3.4 | |
| FSC threshold | 0.143 | | 0.143 | |
| Map resolution range (Å) | 2.5-5.0 | | 2.8-6.5 | |
| **Refinement** |  |  |  |  |
| Model resolution (Å) | 3.1 | | 3.6 | |
| FSC threshold | 0.5 |  | 0.5 | |
| Model resolution range (Å) | 2.5-5.0 | | 2.8-6.5 | |
| Map sharpening B factor (Å^2^) | 92.0 | | 120.7 | |
| Model composition |  |  |  |  |
| Non-hydrogen atoms | 7,317 | | 6,925 | |
| Protein residues | 857 | | 835 | |
| Ligands | 16 | | 4 | |
| B factors (Å^2^) |  |  |  |  |
| Protein | 24.5 | | 76.5 | |
| Ligand | 30.7 | | 109.06 | |
| R.m.s. deviations |  |  |  |  |
| Bond lengths (Å) | 0.003 | | 0.003 | |
| Bond angles (°) | 0.520 | | 0.533 | |
| Validation |  |  |  |  |
| Molprobity score | 1.69 | | 1.62 | |
| Clash score | 7.29 | | 8.11 | |
| Poor rotamers (%) | 0.25 | | 0.00 | |
| Rama-Z |  | |  | |
| Whole | 1.17 | | 0.43 | |
| Helix | 1.59 | | 1.11 | |
| Sheet | 0.74 | | 0.55 | |
| loop | 0.09 | | -0.50 | |
| Ramachandran plot |  |  |  |  |
| Favored (%) | 95.84 | | 96.96 | |
| Allowed (%) | 4.16 | | 3.04 | |
| Disallowed (%) | 0 | | 0 | |
| Model vs. Data |  | |  | |
| CC (mask) | 0.83 | | 0.76 | |
| CC (box) | 0.73 | | 0.69 | |
| CC (peaks) | 0.69 | | 0.61 | |
| CC (volume) | 0.79 | | 0.73 | |


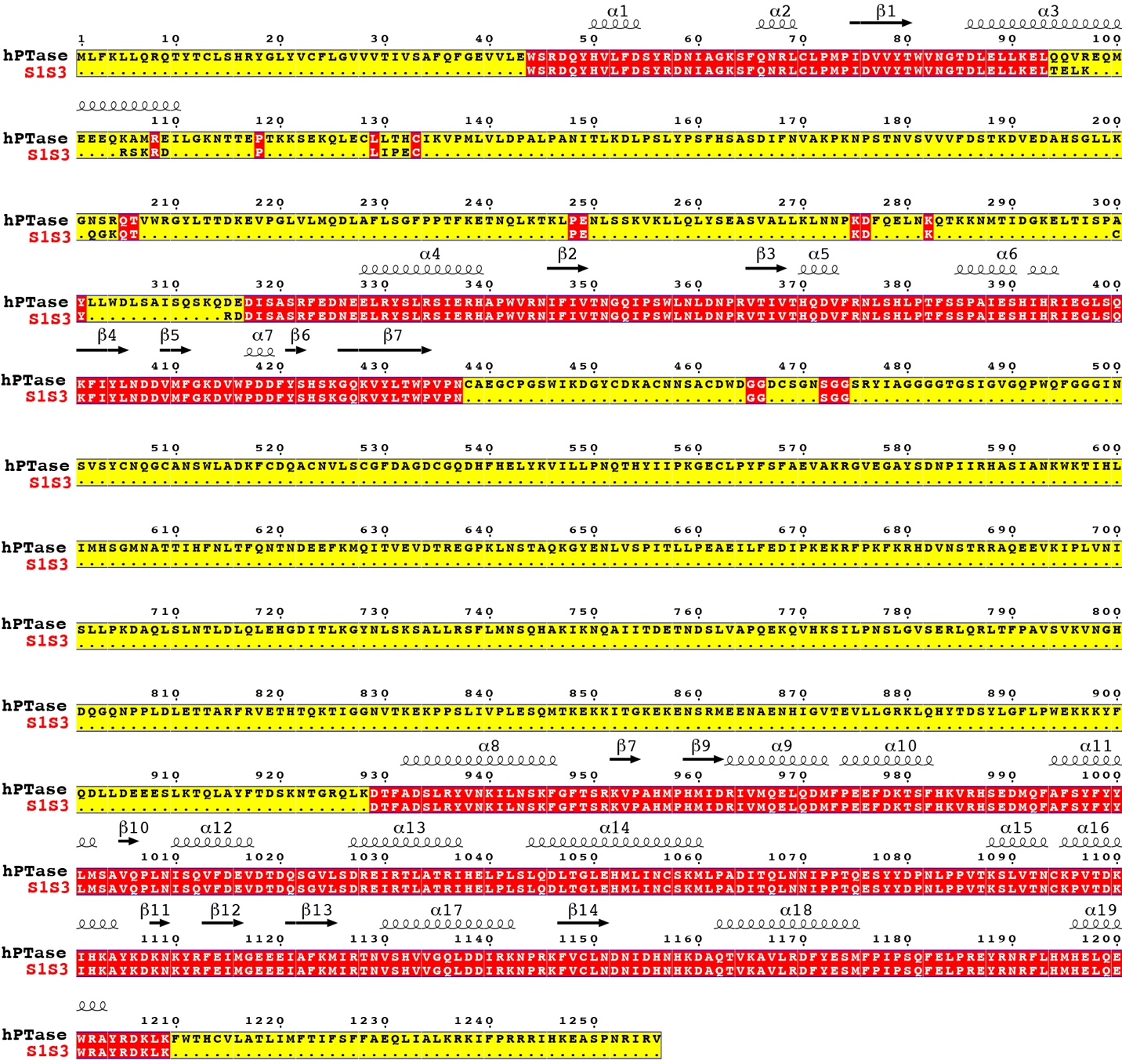


**Figure S1.** **Sequence alignment of the full length human PTase-αβ and the truncated construct S1S3.** The secondary structures are shown and labeled above the sequence.


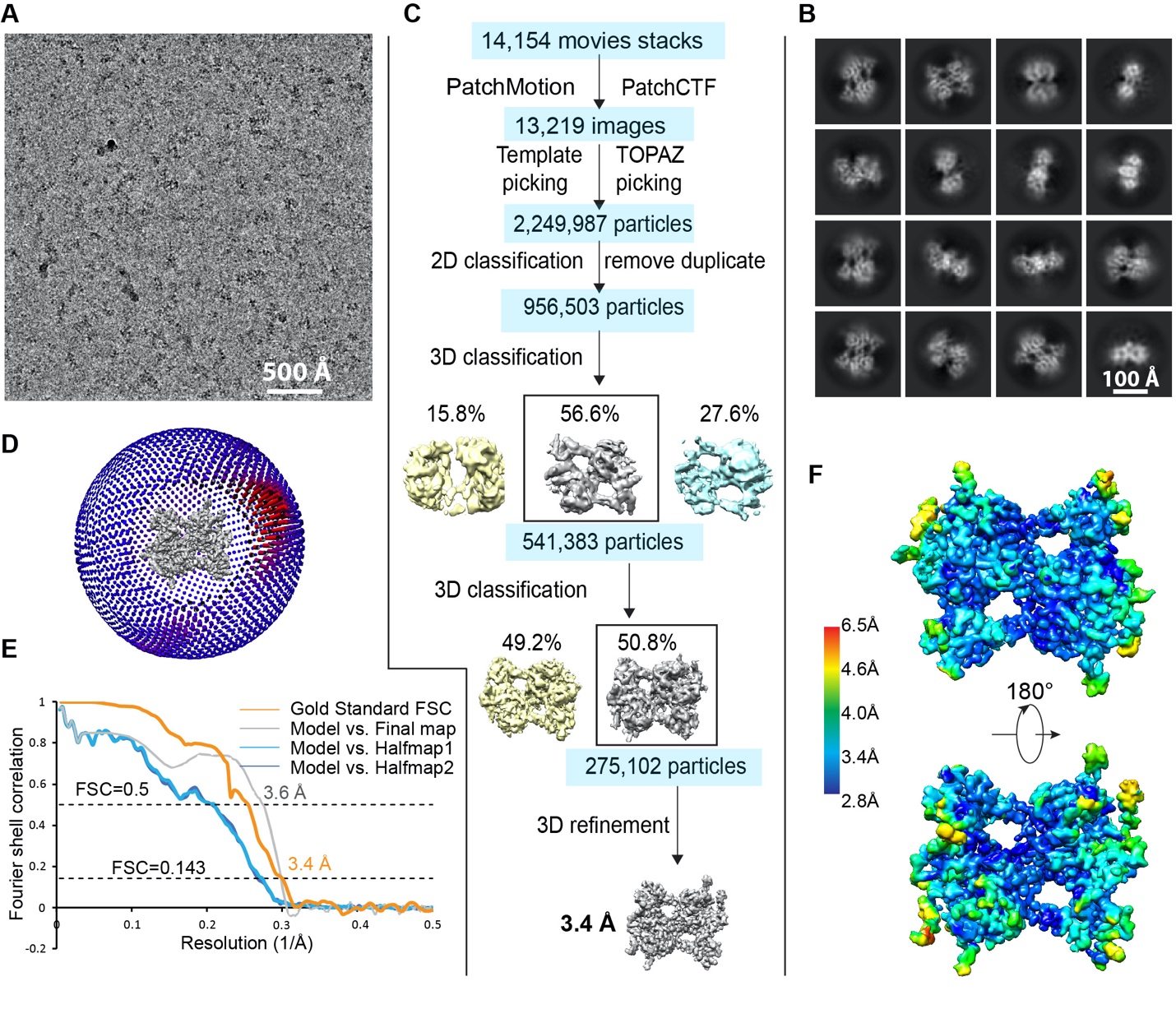


**Figure S2. Data processing of the S1S3 cryo-EM images.** A, A representative micrograph. A total of 13,219 such micrographs were recorded. B, Selected 2D class averages. C, Workflow of data processing from acquired movies to the final 3D map. D, Angular distribution of the final particles used for 3D reconstruction and refinement. E, Gold standard Fourier shell correlation (FSC) of two independently reconstructed two half-maps indicates an overall resolution of 3.4 Å at the correlation 0.143 threshold. Correlations of the atomic model with the final map and with the two halfmaps indicate the atomic model was not over refined. F, Two views of the final EM map surfaced rendered and color coded by the estimated local resolution.


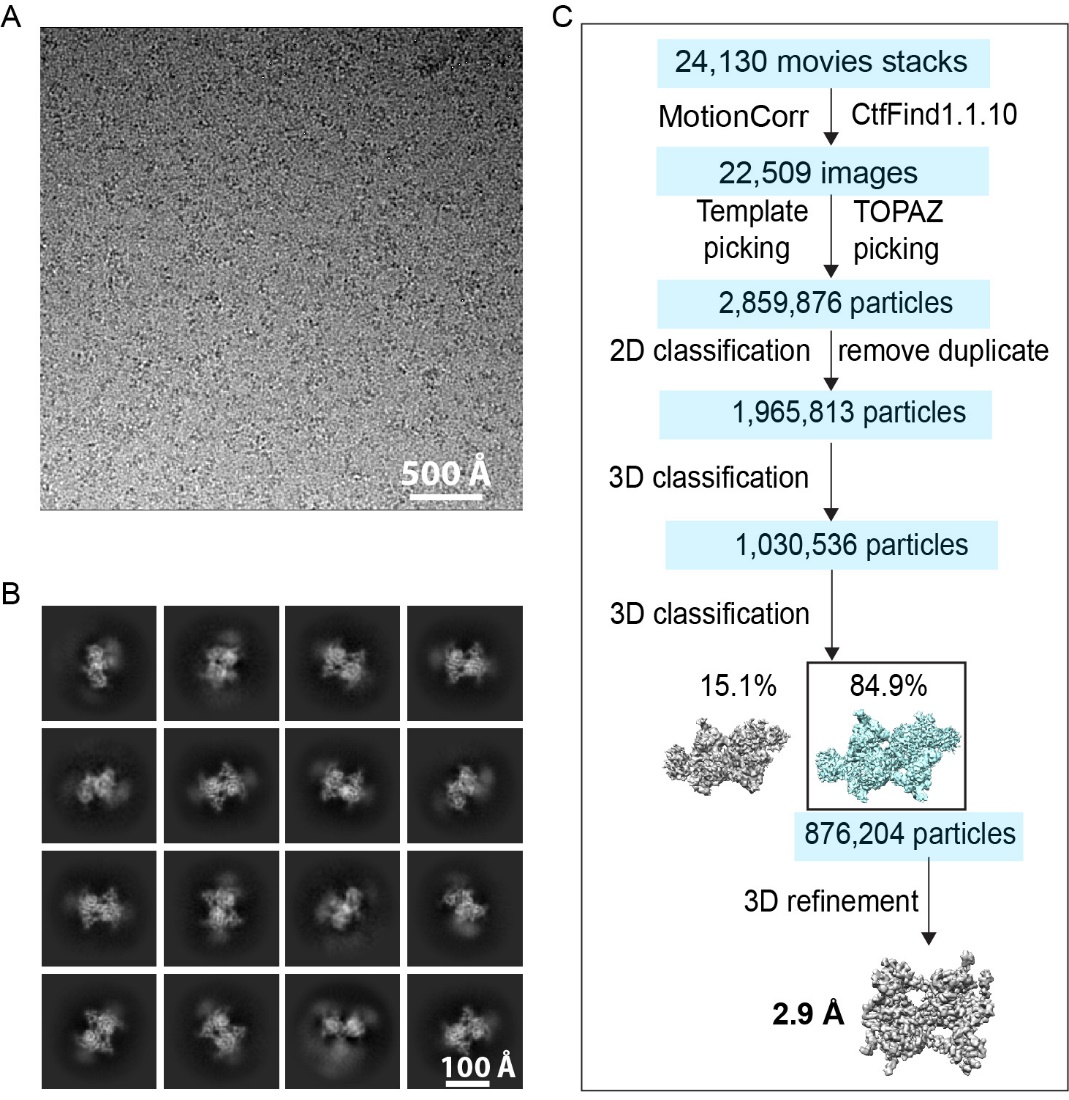


**Figure S3. Processing procedure of cryo-EM data of the UDP-GlcNAc bound hPTase-αβ complex.** A, A representative raw micrograph. A total of 22,509 such micrographs were recorded. B, Selected 2D class averages. C, The work-flow of data processing from acquired movies to the final 3D map.


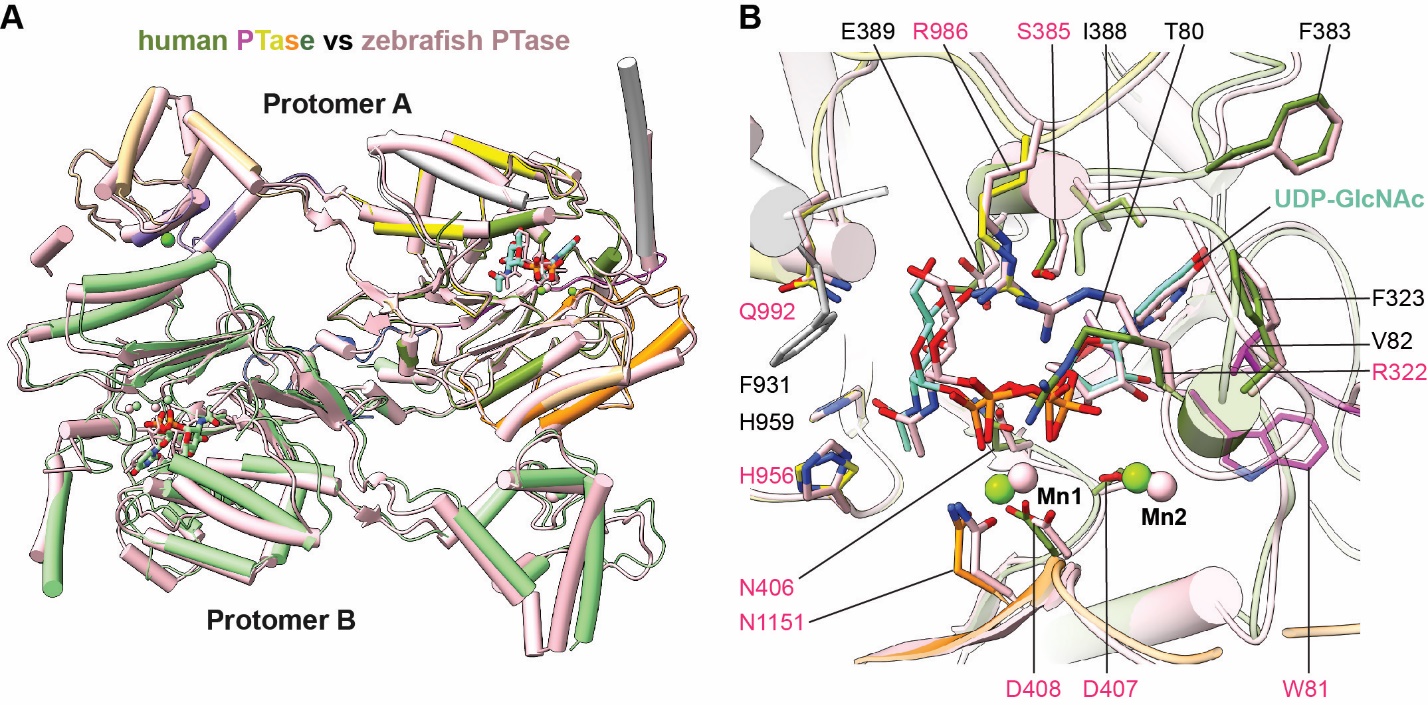


**Figure S4. Structural comparison of the human PTase (hPTase) and zebrafish PTase (zPTase) both bound to UDP-GlcNAc.** A, Superposition of the structures of human PTase (this study) and zebrafish PTase (PDB ID 7SJ2). hPTase is colored the same as in Fig. 3, while the zPTase structure is colored in pink. GlcNAc and bound ions are shown in sticks and spheres, respectively. B, Closed-up view of the UDP-GlcNAc binding pocket. The donor binding residues in hPTase are labeled. Note that residues lining the catalytic pocket adopt nearly identical orientations in the two structures.


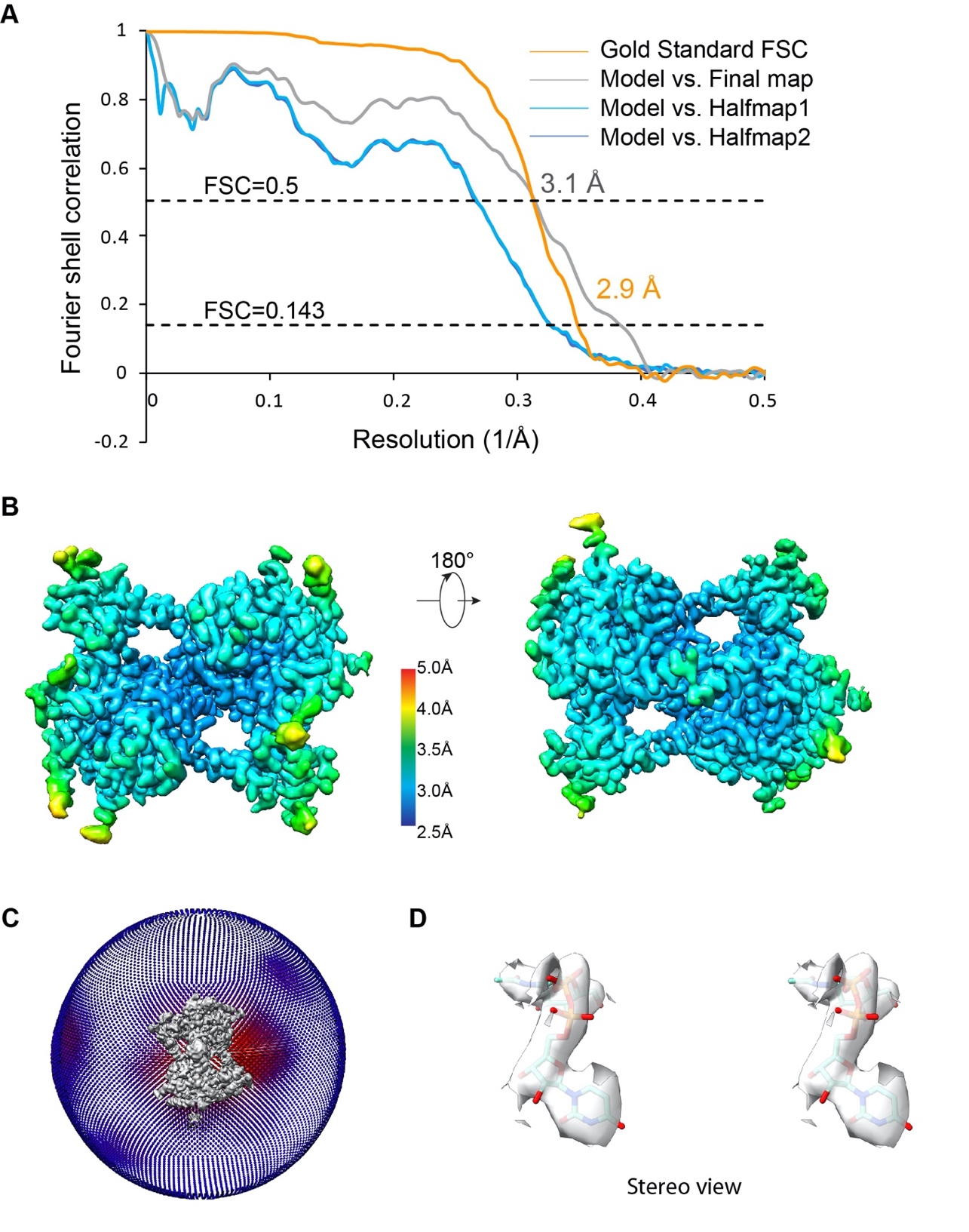


**Figure S5. Characterization of the cryo-EM map of hPTase**-αβ **bound to UDP-GlcNAc.** A, Gold standard Fourier shell correlation (FSC) of the two independently reconstructed halfmaps (halfmap1 and halfmap2) indicates an overall resolution of 2.9 Å at the 0.143 correlation threshold. Correlations of the atomic model with the final map and two half maps indicate the model was not over refined. B, 3D EM map was surface rendered, and color coded by estimated local resolution, and shown in two views. C, Angular distribution of the final particle images used for 3D map reconstruction and refinement. D, The EM density of UDP-GlcNAc superimposed with the atomic model and shown as stereo views.

**
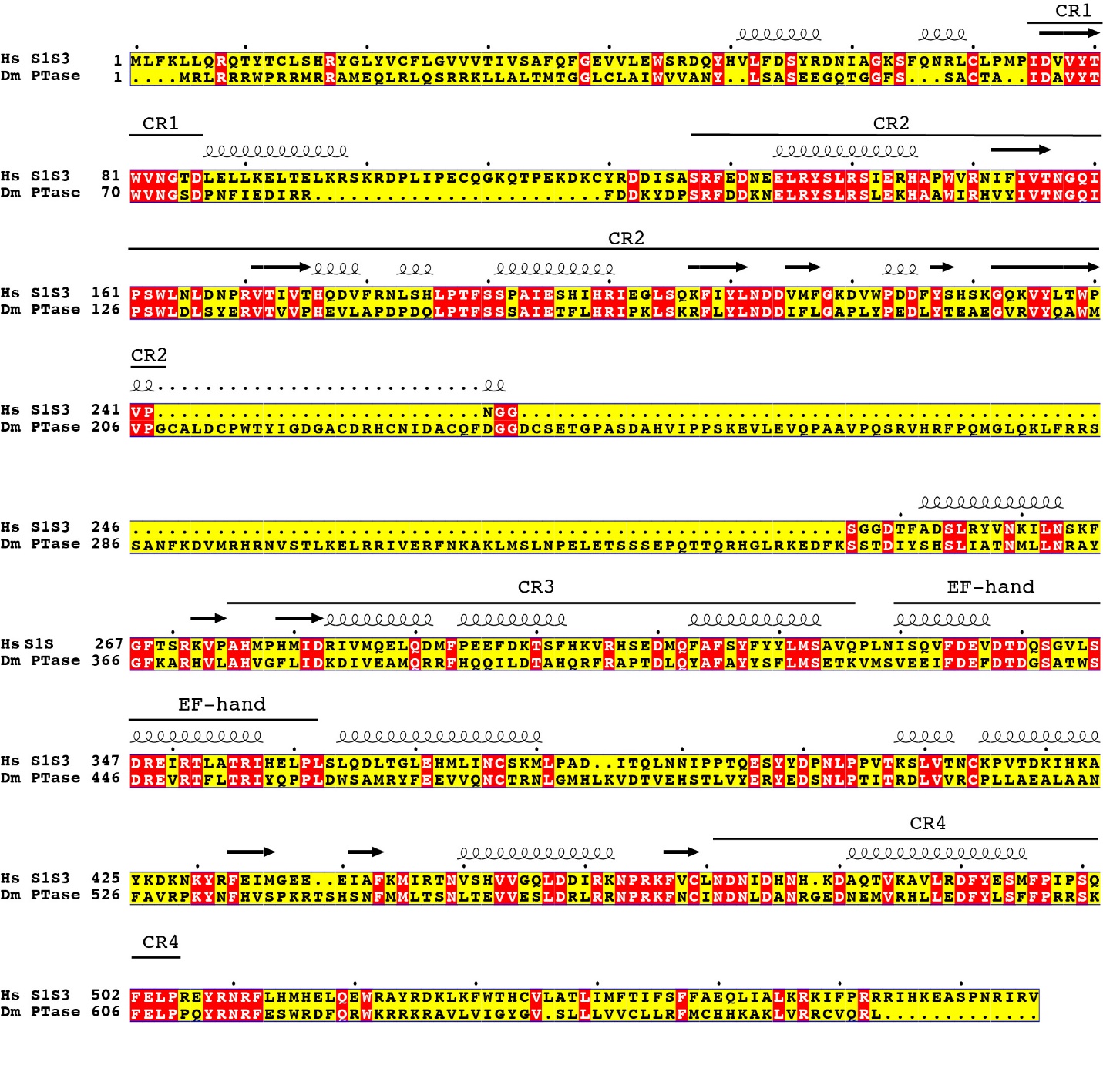
**

**Figure S6.** **Sequence alignment of S1S3 and the *D. melanogaster* PTase.** The secondary structure elements are shown above the sequences, and the conserved regions 1-4 (CR1-4) and the EF-hand are marked by black lines.

**
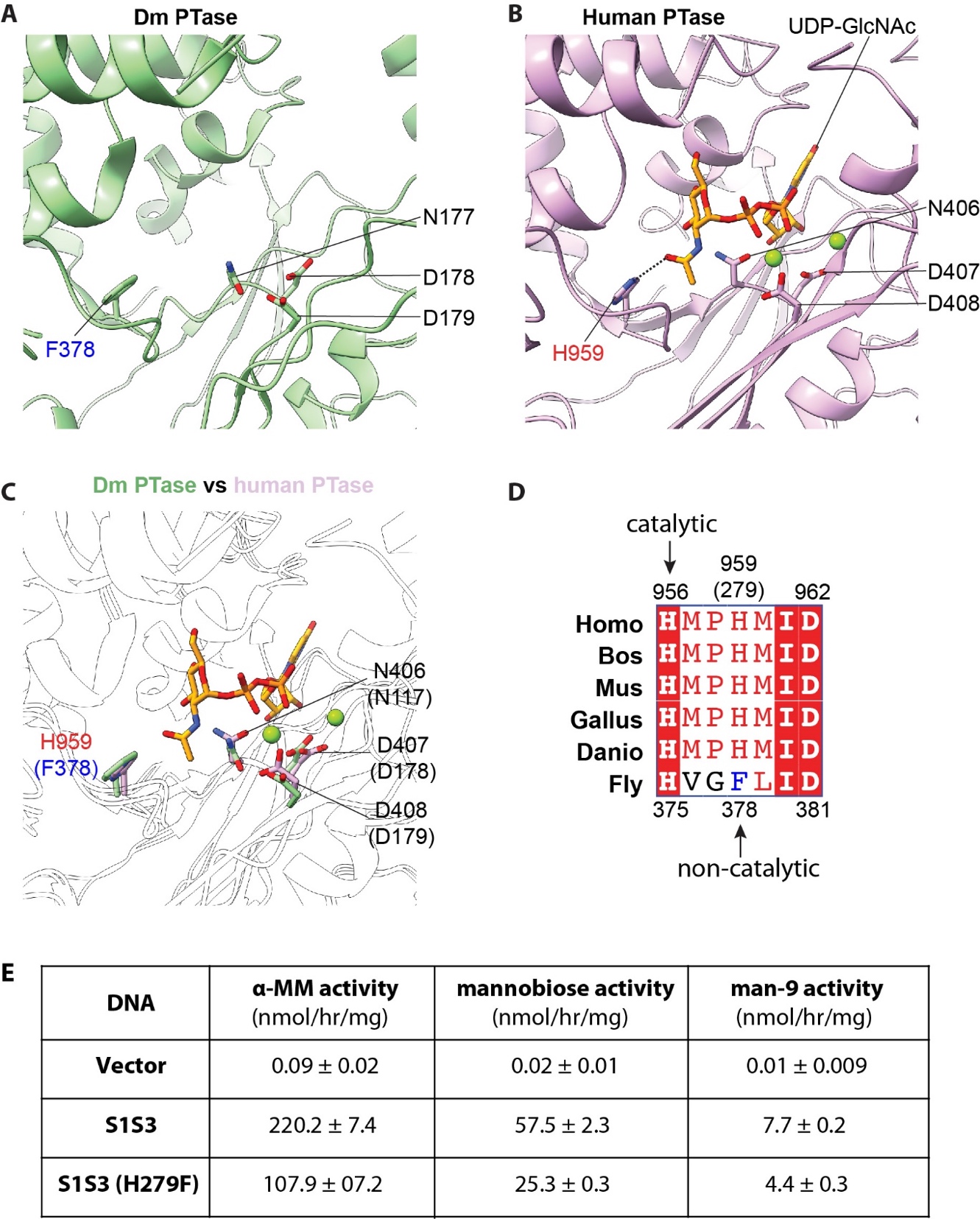
**

**Figure S7.** **The UDP-GlcNAc binding pocket and mutation analysis.** A-C, Close-up view of the catalytic pocket of the Dm PTase alone (A), human PTase with UDP-GlcNAc bound to the pocket (B), and their superimposition (C), highlighting the H-bond between His-959 and the donor in human PTase is absent in Dm PTase. D, Sequence alignment of five homologs. Note the catalytic His-956 is invariant, but the non-catalytic His-959 (His-279 in S1S3) is replaced by Phe-378 in fly. E, PTase activity of S1S3 versus S1S3(H279F) using α-MM, mannobiose, and Man-9 as the acceptor. The data shown are the mean ± SD for 3 independent assays.
